# Supplementary material for: Potassium fertilization enhances both cereal yield and soil organic carbon: a meta-analysis
Source: Nat Commun. 2026 Mar 27;17:4521. doi: 10.1038/s41467-026-71154-z (PMC13195153; doi:10.1038/s41467-026-71154-z)
Supplement: Supplementary file 1 — Supplementary Information [file 41467_2026_71154_MOESM1_ESM.pdf]

Supplementary Material for

Potassium fertilization enhances both cereal yield and soil organic carbon: a meta-analysis

Guopeng Liang[1*], William H. Schlesinger[2*]
[1]Department of Ecology & Evolutionary Biology, Yale University, New Haven, CT, USA
[2]Cary Institute of Ecosystem Studies, Millbrook, NY, USA
*Corresponding author: Guopeng Liang (Guopeng.Liang@yale.edu); William H. Schlesinger (schlesingerw@caryinstitute.org)

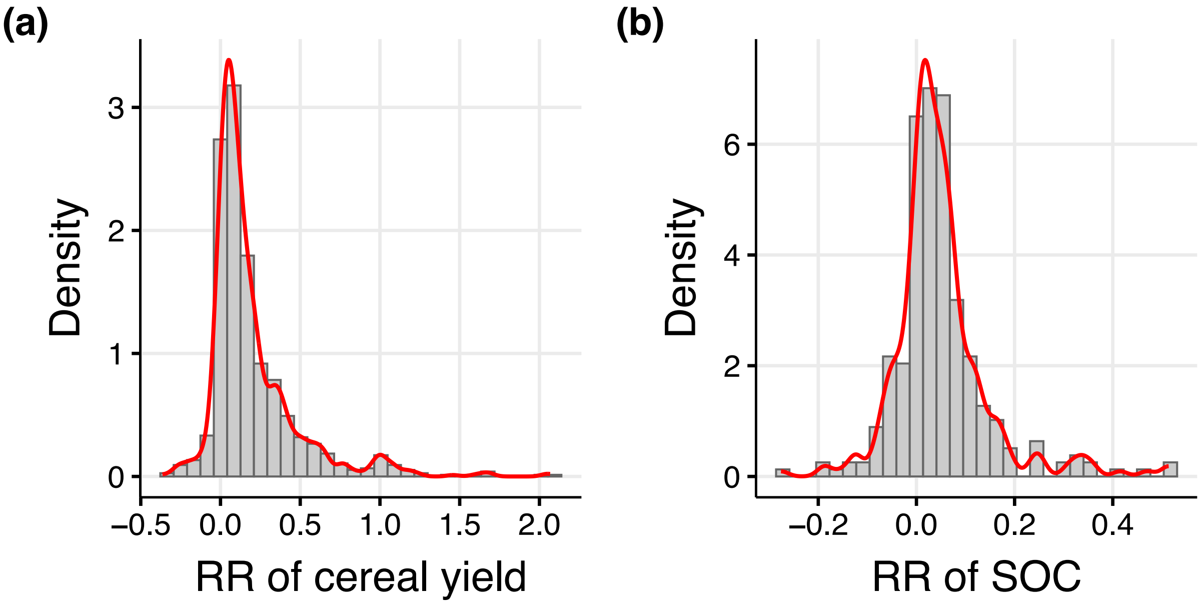

Supplementary Figure 1 Density distributions of the response ratios (RR) for cereal yield (a) and soil organic carbon (SOC, b). Source data are provided as a Source Data file.

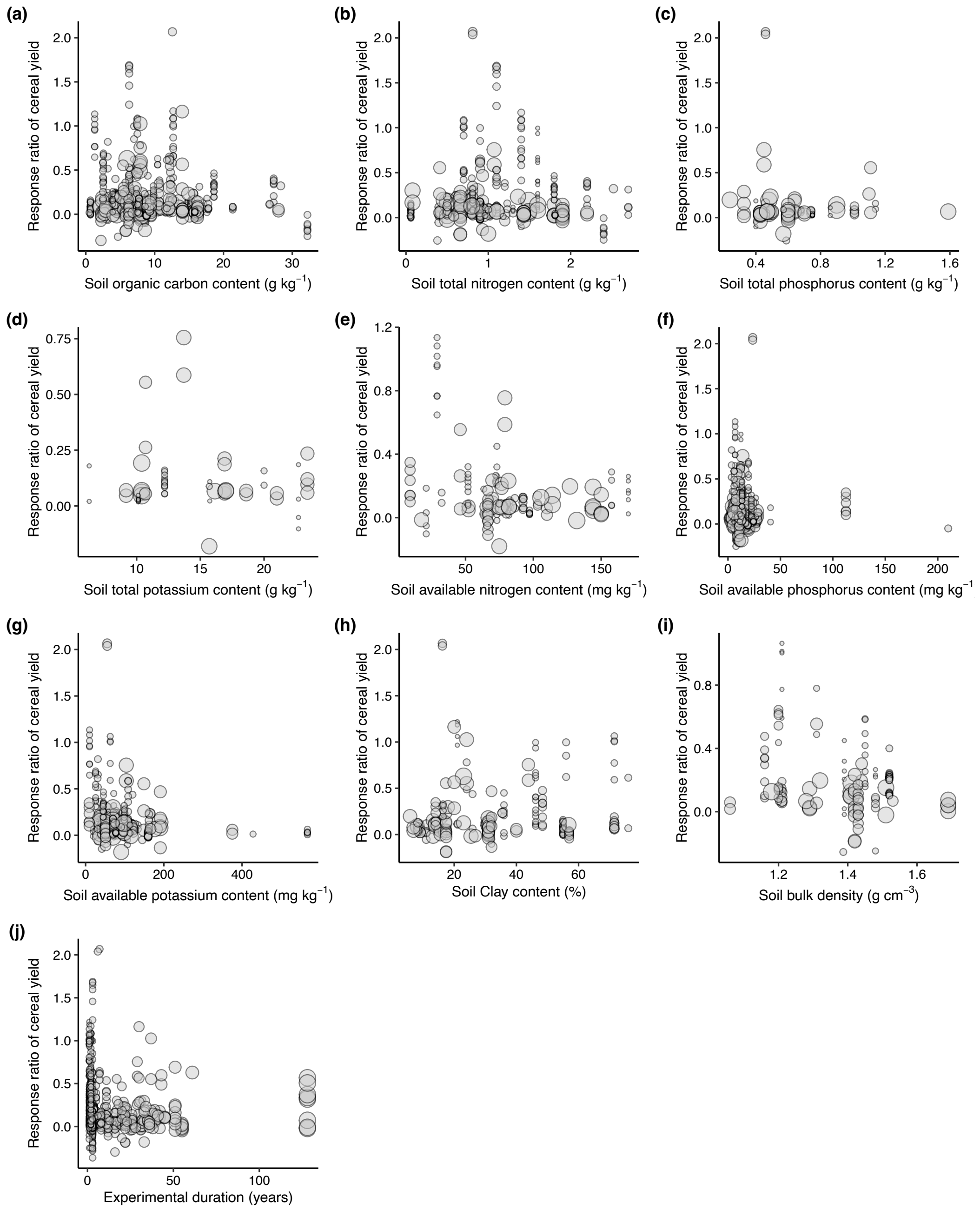

Supplementary Figure 2 Relationships between the response ratio (RR) of cereal yield to potassium fertilization and the predictor variables. (a) Soil organic carbon content, (b) soil total nitrogen content, (c) soil total phosphorus content, (d) soil total potassium content, (e) soil available nitrogen content, (f) soil available phosphorus content, (g) soil available potassium content, (h) soil clay content, (i) soil bulk density, and (j) experimental duration. Circle sizes denote the statistical weight of individual studies. Statistical significance was evaluated using a random-effects meta-regression model, and the significance of regression coefficients was determined using a two-sided Z-test ($P < 0.05$) without adjustments for multiple comparisons. All relationships shown are non-significant ($P > 0.05$). Source data are provided as a Source Data file.

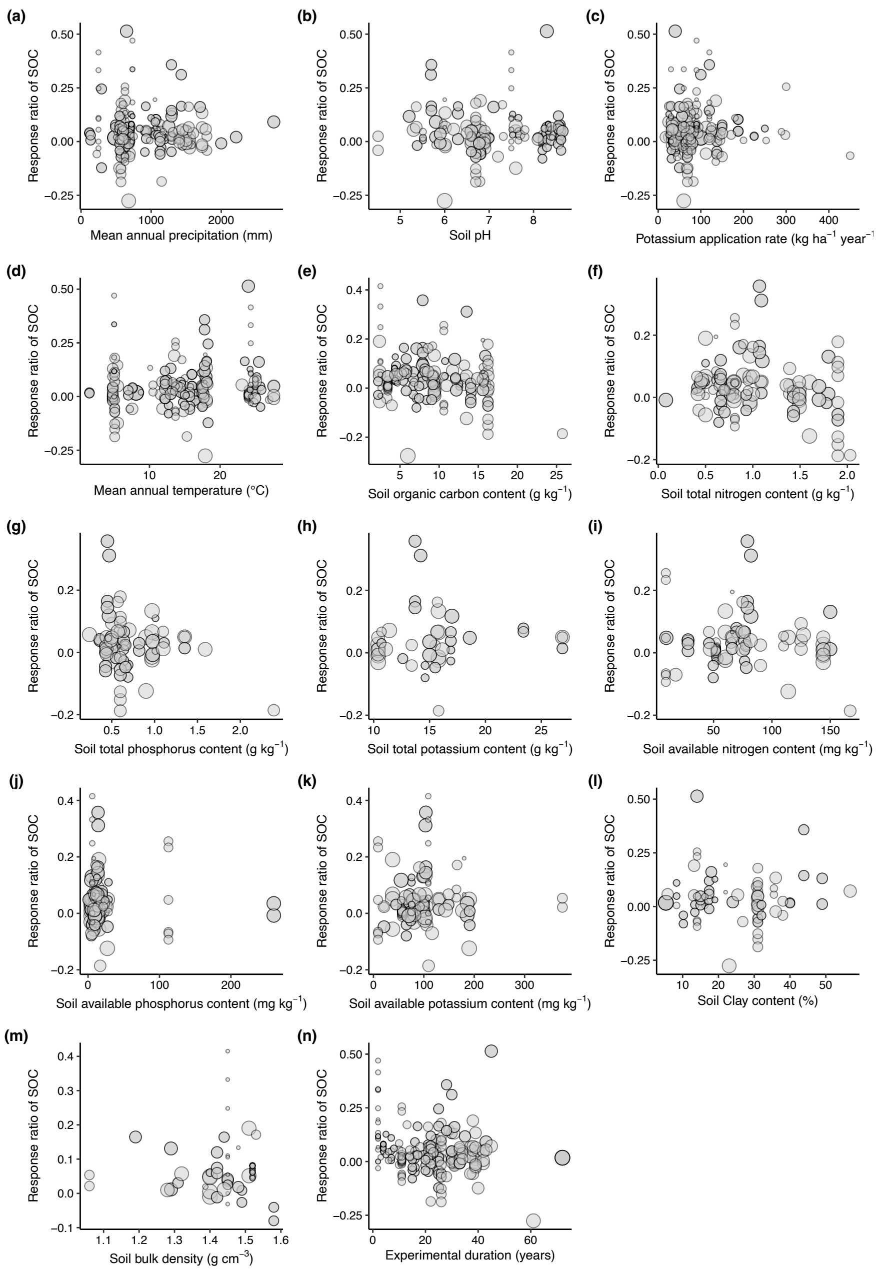

Supplementary Figure 3 Relationships between the response ratio (RR) of soil organic carbon (SOC) to potassium fertilization and the predictor variables. (a) Mean annual precipitation, (b) soil pH, (c) potassium application rate, (d) mean annual temperature, (e) soil organic carbon content, (f) soil total nitrogen content, (g) soil total phosphorus content, (h) soil total potassium content, (i) soil available nitrogen content, (j) soil available phosphorus content, (k) soil available potassium content, (l) soil clay content, (m) soil bulk density, and (n) experimental duration. Circle sizes denote the weight of individual studies. Circle sizes denote the statistical weight of individual studies. Statistical significance was evaluated using a random-effects meta-regression model, and the significance of regression coefficients was determined using a two-sided Z-test ($P < 0.05$) without adjustments for multiple comparisons. All relationships shown are non-significant ($P > 0.05$). Source data are provided as a Source Data file.

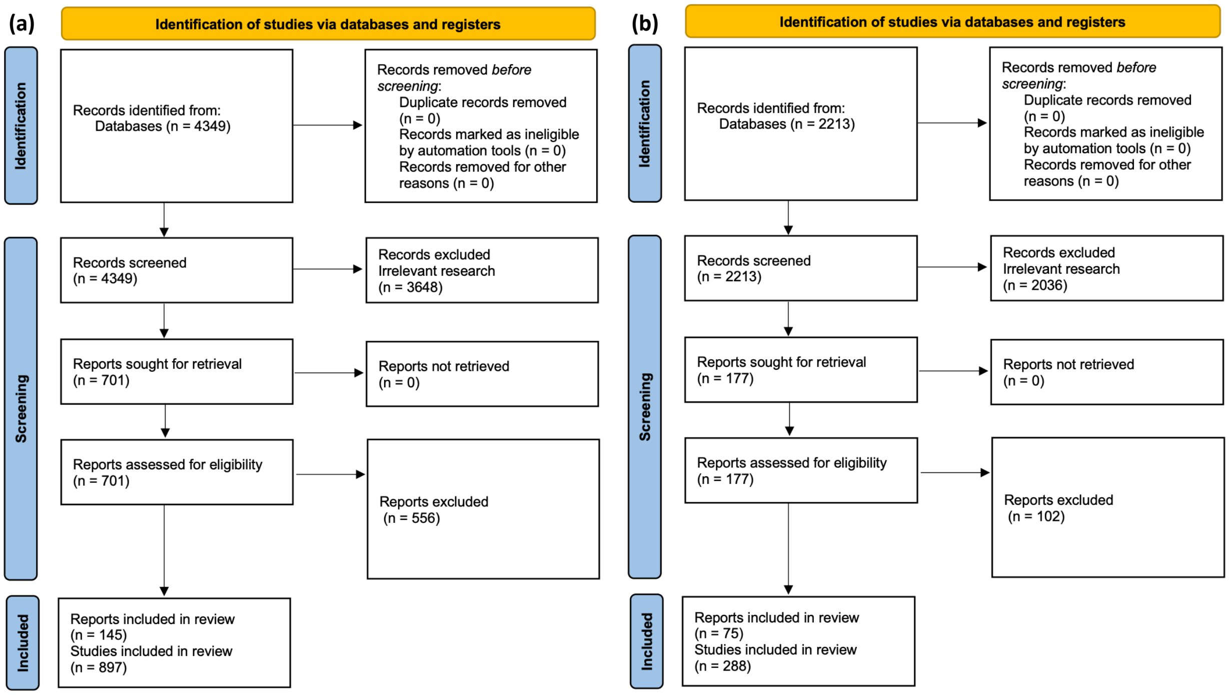

Supplementary Figure 4 PRISMA flow diagram showing the procedure used for the selection of studies for cereal yield (a) and soil organic carbon (b).

(a)

(b)

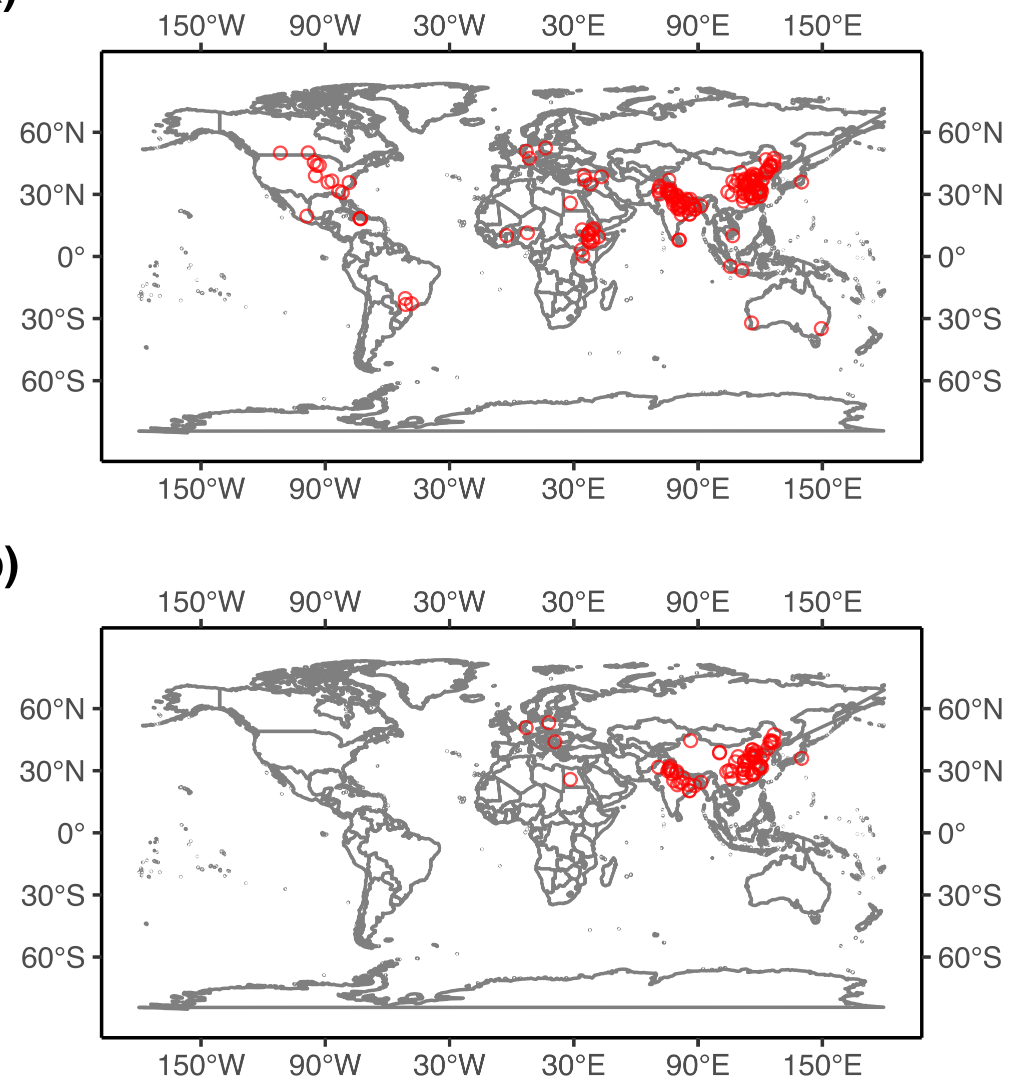

Supplementary Figure 5 Location of potassium fertilization experiments about crop yield (a) and soil organic carbon (b) included in the present study. The underlying world map was generated using the borders function in R (via the ggplot2 and maps packages), with coastline data from the Public Domain; no specific permissions are required for its publication. Source data are provided as a Source Data file.

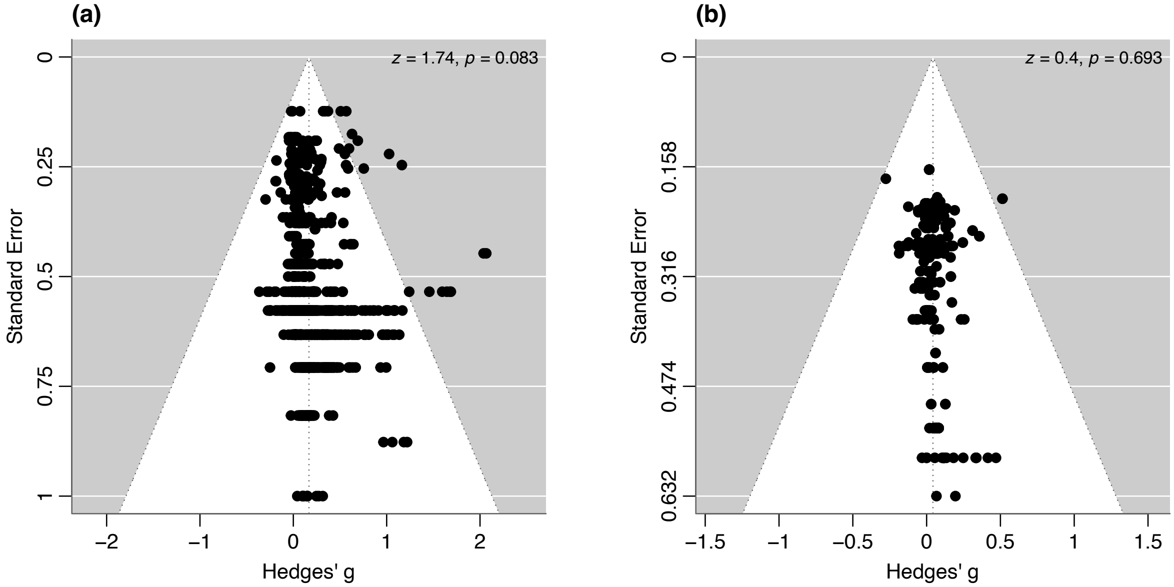

Supplementary Figure 6 Funnel plots for potassium fertilization effects on cereal yield (a) and soil organic carbon (b). Source data are provided as a Source Data file.

Supplementary Table 1 Summary of site characteristics

Parameter	Unit	Cereal yield (*N* = 897)				Soil organic carbon (*N* = 288)			
		Minimum	Maximum	Mean	*N*	Minimum	Maximum	Mean	*N*
Latitude	º	-34.8	52.4	27.5	763	20.3	53.2	33.5	286
Longitude	º	-111.7	149.5	63.7	763	6.7	140.0	100.7	286
MAT	ºC	-0.5	32.1	17.4	757	1.5	27.6	16.2	288
MAP	mm yr^{-1}	61.0	2750.0	994.9	757	120.0	2750.0	866.4	288
Clay	%	5.8	76.0	34.7	199	5.3	56.8	23.5	104
ISOC	g kg^{-1}	0.5	32.2	9.3	542	2.2	25.7	8.4	231
TN	g kg^{-1}	0.05	2.70	1.07	343	0.08	2.03	1.01	148
TP	g kg^{-1}	0.24	1.59	0.63	97	0.24	2.39	0.68	106
TK	g kg^{-1}	6.3	23.4	14.5	59	10.4	26.9	15.7	64
AN	mg kg^{-1}	9.1	170.0	80.2	128	9.1	167.1	70.9	109
AP	mg kg^{-1}	1.9	210.0	15.1	389	1.9	260.0	20.9	169
AK	mg kg^{-1}	9.2	567.0	92.8	299	9.2	375.0	93.5	141
BD	g cm^{-3}	1.06	1.69	1.38	124	1.06	1.58	1.44	82
pH		4.7	8.8	6.9	594	4.5	8.7	7.3	215
Duration	yr	1	128	9	873	2	72	21	281
K rate	kg ha^{-1} yr^{-1}	10	450	83	864	18	450	81	274

N: study number; MAT: mean annual temperature; MAP: mean annual precipitation; Clay: initial soil clay content; ISOC: initial soil organic carbon; TN: initial soil total nitrogen content; TP: initial soil total phosphorus content; TK: initial soil total potassium content; AN: initial soil available nitrogen content; AP: initial soil available phosphorus content; AK: initial soil available potassium content; BD: initial soil bulk density; pH: initial soil pH; Duration: experimental duration; K rate: potassium application rate.
