# Supplementary material for: Potassium fertilization enhances both cereal yield and soil organic carbon: a meta-analysis
Source: Nat Commun. 2026 Mar 27;17:4521. doi: 10.1038/s41467-026-71154-z (PMC13195153; doi:10.1038/s41467-026-71154-z)
Supplement: Supplementary file 3 — Description of Additional Supplementary Files [file 41467_2026_71154_MOESM3_ESM.pdf]

Description of Additional Supplementary Files

File Name: Supplementary Data 1

Description: A list of the papers included in the meta-analysis
