# Supplementary material for: Potassium fertilization enhances both cereal yield and soil organic carbon: a meta-analysis
Source: Nat Commun. 2026 Mar 27;17:4521. doi: 10.1038/s41467-026-71154-z (PMC13195153; doi:10.1038/s41467-026-71154-z)
Supplement: Supplementary file 5 — Reporting Summary [file 41467_2026_71154_MOESM5_ESM.pdf]

nature portfolio

Corresponding author(s): Guopeng Liang, William H. Schlesinger

Last updated by author(s): 03/03/2026

Reporting Summary

Nature Portfolio wishes to improve the reproducibility of the work that we publish. This form provides structure for consistency and transparency in reporting. For further information on Nature Portfolio policies, see our Editorial Policies and the Editorial Policy Checklist.

Statistics

For all statistical analyses, confirm that the following items are present in the figure legend, table legend, main text, or Methods section.

n/a	Confirmed	
☐	☒	The exact sample size (*n*) for each experimental group/condition, given as a discrete number and unit of measurement
☐	☒	A statement on whether measurements were taken from distinct samples or whether the same sample was measured repeatedly
☐	☒	The statistical test(s) used AND whether they are one- or two-sided<br>*Only common tests should be described solely by name; describe more complex techniques in the Methods section.*
☐	☒	A description of all covariates tested
☐	☒	A description of any assumptions or corrections, such as tests of normality and adjustment for multiple comparisons
☐	☒	A full description of the statistical parameters including central tendency (e.g. means) or other basic estimates (e.g. regression coefficient) AND variation (e.g. standard deviation) or associated estimates of uncertainty (e.g. confidence intervals)
☐	☒	For null hypothesis testing, the test statistic (e.g. *F*, *t*, *r*) with confidence intervals, effect sizes, degrees of freedom and *P* value noted<br>*Give P values as exact values whenever suitable.*
☒	☐	For Bayesian analysis, information on the choice of priors and Markov chain Monte Carlo settings
☒	☐	For hierarchical and complex designs, identification of the appropriate level for tests and full reporting of outcomes
☐	☒	Estimates of effect sizes (e.g. Cohen's *d*, Pearson's *r*), indicating how they were calculated

Our web collection on statistics for biologists contains articles on many of the points above.

Software and code

Policy information about availability of computer code

Data collection	Engauge Digitizer software (Free Software Foundation, Boston, MA, USA; Version 12.1) was used to extract the data in figures.
Data analysis	All statistical analyses and graphs were conducted in RStudio (Version 2024.04.0+735), using packages including terra, geodata, metafor, patchwork, cowplot, gbm, broom, tidyverse, sf, dplyr, rnaturalearth, rnaturalearthdata, purrr, ggplot2, and stringr. The R script needed to reproduce the results are archived in in Zenodo at https://doi.org/10.5281/zenodo.18839011.

For manuscripts utilizing custom algorithms or software that are central to the research but not yet described in published literature, software must be made available to editors and reviewers. We strongly encourage code deposition in a community repository (e.g. GitHub). See the Nature Portfolio guidelines for submitting code & software for further information.

Data

Policy information about availability of data

All manuscripts must include a data availability statement. This statement should provide the following information, where applicable:

- Accession codes, unique identifiers, or web links for publicly available datasets
- A description of any restrictions on data availability
- For clinical datasets or third party data, please ensure that the statement adheres to our policy

The datasets that support the findings of this study are openly available in Zenodo at https://doi.org/10.5281/zenodo.1883901132. Source data are provided with this paper.

April 2023

Research involving human participants, their data, or biological material

Policy information about studies with human participants or human data. See also policy information about sex, gender (identity/presentation), and sexual orientation and race, ethnicity and racism.

Reporting on sex and gender	NA
Reporting on race, ethnicity, or other socially relevant groupings	NA
Population characteristics	NA
Recruitment	NA
Ethics oversight	NA

Note that full information on the approval of the study protocol must also be provided in the manuscript.

Field-specific reporting

Please select the one below that is the best fit for your research. If you are not sure, read the appropriate sections before making your selection.

☐ Life sciences ☐ Behavioural & social sciences ☒ Ecological, evolutionary & environmental sciences

For a reference copy of the document with all sections, see nature.com/documents/nr-reporting-summary-flat.pdf

Ecological, evolutionary & environmental sciences study design

All studies must disclose on these points even when the disclosure is negative.

Study description	This is a meta-analysis determining the effects of potassium fertilization on cereal yield and soil organic carbon.
Research sample	897 and 288 paired observations were collected to assess potassium effects on cereal yield and soil organic carbon, respectively.
Sampling strategy	If the sample size was not provided in papers, it was assigned as the median of the sample size of the other studies, which was 3 for both cereal yield and soil organic carbon.
Data collection	We used the Web of Science to conduct a literature search in November 2025. Keywords "potassium fertilizer AND crop yield" and "potassium fertilizer AND soil carbon" were used to search for papers reporting K effects on cereal yield and SOC, respectively.
Timing and spatial scale	The publications included in the dataset ranged from 1992 to 2025. The observations were distributed across the globe.
Data exclusions	The following criteria were used to select papers: (1) studies were conducted at field sites in agricultural ecosystems; (2) to remove the potential effects of N and P, the difference between K treatment and control should derive only from K addition (e.g., K vs. control; NK vs. N; PK vs. P; NPK vs. NP; and organic fertilizer [O] +K [OK] vs. O); (3) only yield of cereal, which plays a great role in global food security, was included in the present study; (4) if SOC and other soil parameters were reported at multiple soil depths, the data in surface soils (within 20cm depth) were used; (5) for studies reporting results at multiple time points, only the data at the last time point was included; and (6) cereal yield or SOC from different crop types, tillage systems, or K application rates in the same study were regarded as independent observations.
Reproducibility	All attempts to repeat the analysis and results were successful.
Randomization	NA
Blinding	All attempts to repeat the analysis and results were successful.

Did the study involve field work? ☐ Yes ☒ No

Reporting for specific materials, systems and methods

We require information from authors about some types of materials, experimental systems and methods used in many studies. Here, indicate whether each material, system or method listed is relevant to your study. If you are not sure if a list item applies to your research, read the appropriate section before selecting a response.

Materials & experimental systems

n/a	Involved in the study
☒	☐ Antibodies
☒	☐ Eukaryotic cell lines
☒	☐ Palaeontology and archaeology
☒	☐ Animals and other organisms
☒	☐ Clinical data
☒	☐ Dual use research of concern
☒	☐ Plants

Methods

n/a	Involved in the study
☒	☐ ChIP-seq
☒	☐ Flow cytometry
☒	☐ MRI-based neuroimaging

Plants

Seed stocks	NA
Novel plant genotypes	NA
Authentication	NA
